# Epithelioid Hemangioendothelioma: Incidence, Mortality, Prognostic Factors, and Survival Analysis Using the Surveillance, Epidemiology, and End Results Database

**DOI:** 10.1155/2022/2349991

**Published:** 2022-09-16

**Authors:** Zhen Liu, Shuting He

**Affiliations:** ^1^Department of Burns and Plastic Surgery, Beijing Children's Hospital, Capital Medical University, National Center for Children's Health, Beijing 100034, China; ^2^Department of Anesthesiology, Peking University First Hospital, Beijing 100034, China

## Abstract

**Background:**

Although epithelioid hemangioendothelioma (EHE) is a rare and aggressive vascular tumor, its demographic characteristics remain unclear. We used the surveillance, epidemiology, and end results (SEER) database to determine the clinical features, incidence, and prognostic factors associated with overall survival in patients with EHE.

**Methods:**

The demographic and clinical data of patients with EHE were extracted from the SEER database (1975-2019) to calculate the incidence of EHE and survival rate in these patients. The Cox proportional hazards model and Kaplan-Meier method were used to analyze the prognostic factors of overall survival in these patients. A nomogram and time-dependent receiver operating characteristic (ROC) curve were employed to predict the 3- and 5-year survival rate.

**Results:**

The overall incidence rate (IR) of EHE was 0.230 (95%confidence interval [CI] = 0.201–0.263) per 1,000,000 person-years. According to the age-stratified IR, the highest age-adjusted IR was observed in patients aged 60–79 years (0.524 per 1,000,000 person-years, 95%CI = 0.406–0.665). The majority (30.8%) of the tumors were located in the soft tissue and skin, followed by lesions in the abdomen (28%), respiratory system (19%), bone and joint (8.6%), and others. The 5-year overall survival rate was 55.6% (95%CI = 32.8–73.5%). Multiple Cox regression analysis revealed that age >80 years (hazard ratio [HR] = 8.57, 95%CI = 2.32–31.63, *P* < 0.001), African-American race (HR = 2.52, 95%CI = 1.31–4.85, *P* < 0.01), “American Indian/Alaska Native” or “Asian or Pacific Islander” (HR = 2.99, 95%CI = 1.5–5.96, *P* < 0.01) race, and respiratory tumors (HR = 2.55, 95%CI = 1.37–4.75, *P* < 0.01) were distinctly related to worse overall survival. The calibration plots demonstrated good consistency between nomogram-predicted and actual survival. The area under the time-dependent ROC curve was 0.721 (95%CI = 0.63–0.81) and 0.719 (95%CI = 0.63–0.81) for the 3- and 5-year survival, respectively. For the convenience of researchers and clinicians, we designed an online dynamics nomogram to predict the survival rate.

**Conclusion:**

EHE is a relatively rare vascular tumor, which principally occurs in the soft tissue and skin. It most commonly occurs in patients aged 60–79 years and its incidence has increased in recent years. Age at diagnosis, race, and tumor location may affect the overall survival outcomes.

## 1. Introduction

Epithelioid hemangioendothelioma (EHE) is considered an intermediate or borderline malignant vascular tumor. It was first described in 1982 and involves various organs, including the liver, soft tissues, and bone [1, 2]. Its typical histological features include irregular vasculature, malignant endothelial cell lining, pinocytotic vesicles, and occasional Weibel-Palade bodies [3, 4]. The tumor has an aggressive clinical course, with a tendency for both local recurrence and regional lymph node metastasis. Certain types of the tumor could develop a life-threatening hemoptysis upon invasion into the trachea and pleura [5]. Moreover, EHE is considered as the most common malignant vascular tumor of bone and can easily result in recurrence and metastasis [6, 7]. The overlapping morphologic features make diagnosis and management challenging [8, 9]. The incidence of EHE is approximately one in one million people, making it an extremely uncommon kind of cancer. Due to the fact that it is so uncommon, the majority of the research that has been published so far consists of case reports, with a few retrospective descriptive case series thrown in for good measure. The purpose of these case series is to better characterize the clinical, pathologic, and molecular characteristics, as well as to derive insight into treatment approaches [10, 11]. Currently, no comprehensive study exists on the demographic characteristics and prognostic factors of the overall survival of EHE.

Considering the challenging diagnosis and treatment of EHE, we designed this study using demographic and clinical data from the surveillance, epidemiology, and end results (SEER) database to understand the features of onset and risk factors of prognosis in patients with EHE. The SEER datasets, which contains cancer statistics from roughly twenty-eight percent of the population of the United States, could be considered a relatively large population-based cohort of patients with EHE [12].

## 2. Materials and Methods

### 2.1. Data Collection

The SEER database was searched for the pertinent information on cases diagnosed with EHE from 1975 all the way up till 2019. The variable titles based on the International Classification of Diseases for Oncology (ICD-O-3) histology codes, 9133/1 EHE and 9133/3 EHE, malignant, were considered the diagnosis of EHE. The basic information of these patients including age, race, sex, tumor location, lymph node metastasis, survival time, treatment, SEER cause-specific death classification, and tumor size, was recorded.

Based on the SEER database policy, the overall incidence rate (IR) and survival rate were analyzed using SEER∗Stat software [12]. The rates are per 1,000,000 and age-adjusted to the 2000 US standard population standard, with 95% confidence intervals (CI, Tiwari mod) for the rates and ratios. Moreover, the rates were calculated based on the age at diagnosis, sex, and race. Age was divided into five groups: 0–19, 20–39, 40–59, 60–79, and ≥80 years. The study population was categorized into male and female groups. Race was classified into three groups: white (Caucasians), black (African American), and other (“American Indian/Alaska Native” or “Asian or Pacific Islander”). The IR was considered significantly different when the *P* value is *<*0.05. The tumor location was divided into the following groups: soft tissue and skin, bone and joints, respiratory system, abdomen, head and neck, and others. Treatment regimens were classified into surgery and no surgery groups.

### 2.2. Statistical Analysis

Descriptive statistics were utilized in order to conduct the analysis on the demographic and clinical data. The data were analyzed using R version 3.6.0 (R Foundation for Statistical Computing, Vienna, Austria). The significance of the variables associated with the overall survival was evaluated using the univariate cox proportional hazards model. Multivariate cox regression analysis was subsequently used to analyze the significant variables as independent predictors for the overall survival. The Kaplan–Meier method and log-rank test were used to analyze the survival curves. These results of the multivariate analysis were represented as a nomogram [13]. The performance of the nomogram was evaluated using the concordance index (also known as the C-index), in addition to the calibration curve [14]. Time-dependent ROC curve assays were also used to evaluate the predictive value. Significance was assumed for two-sided *P* values <0.05.

## 3. Results

### 3.1. Patient Characteristics

**Figure 1:**
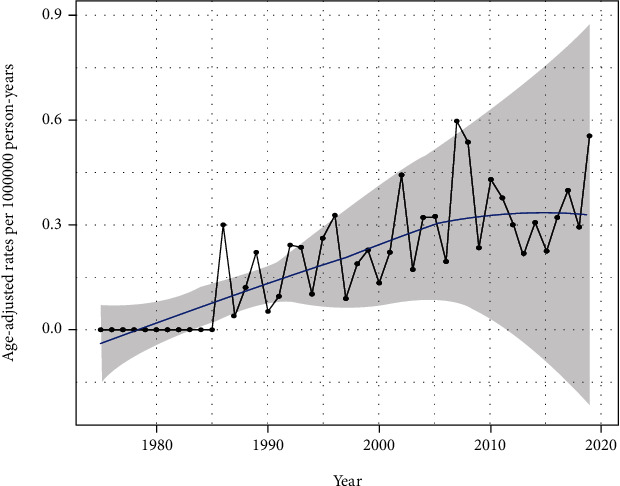
The age-adjusted incidence rate of epithelioid hemangioendothelioma from 1975 to 2019.

**Figure 2:**
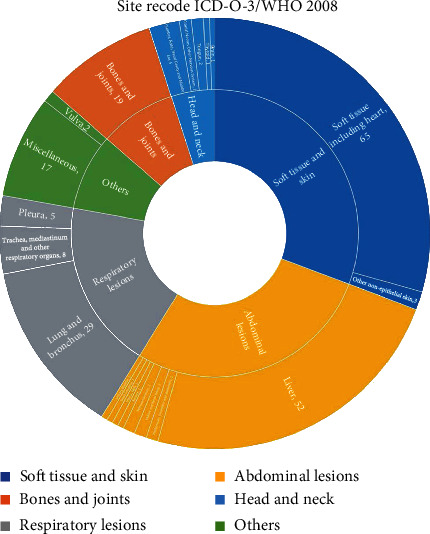
Lesion site and proportion of epithelioid hemangioendothelioma.

**Figure 3:**
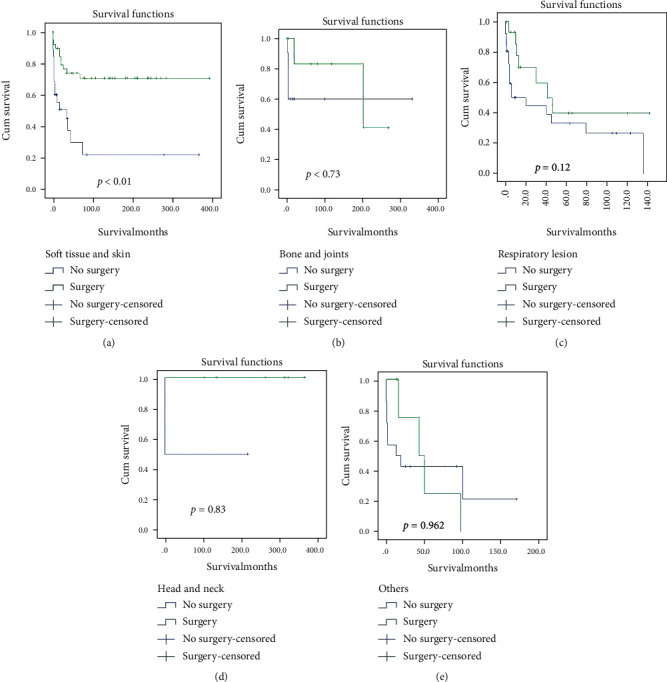
Kaplan-Meier (KM) survival curves in different locations according to the surgery and no surgery groups. (a) KM survival curves in soft tissues and skin. (b) KM survival curves in bone and joints. (c) KM survival curves in respiratory lesions. (d) KM survival curves in head and neck. (e) KM survival curves in other organs.

Following relevant data retrieval, a total of 221 patients were enrolled. The overall IR of EHE was 0.230 per 1,000,000 person-years. The IR of EHE is gradually increasing in recent years in the United States population (Figure 1). According to the age-stratified IR, the highest age-adjusted IR was in patients aged 60–79 years (0.524 per 1,000,000 person-years, 95%CI = 0.406–0.665) followed by patients aged 40–59 years (0.349 per 1,000,000 person-years, 95%CI = 0.278–0.431) (Table 1). Tumors were rarely observed in 0–19-year-old patients, with an age-adjusted IR of 0.036 per 1,000,000 person-years (95%CI = 0.017–0.066). A total of 198 patients, including 98 male and 123 female participants, were enrolled in the study; the sex-stratified IR was 0.212 (95%CI = 0.172–0.259) and 0.246 (95%CI = 0.204–0.293) per 1,000,000 person-years in males and females, respectively. According to the SEER database data, the race-stratified IR was 0.230 (95%CI = 0.200–0.270), 0.272 (95%CI = 0.165–0.421), and 0.164 (95%CI = 0.010–0.260) in Caucasians, African Americans, and other races (American Indian/AK Native, Asian/Pacific Islander), respectively. The IR showed no statistical difference in terms of sex and race. Our results also revealed that EHE was observed in various organs. The majority (30.8%) of tumors were located in the soft tissue and skin, followed by the abdomen (28%), respiratory system (19%), bone and joints (8.6%), head and neck (5%), and other (8.6%) organs, such as the vulva, and other miscellaneous lesions (Figure 2). The liver (24%) and lung and bronchus (13%) are the most commonly affected organs in the abdominal and thoracic cavities, respectively. Among these patients, only 11 had regional lymph node metastasis and 3 patients had distant lymph node metastases. The average diameter of the tumor was 49.90 ± 44.28 mm (median = 39 mm, range 6 − 250 mm). Approximately 47.1% of the patients underwent surgery for EHE. In the operation group, 42.6% lesions were located in the soft tissue and skin, wherein most of the patients (60.3%) had a better overall survival than those in the no surgery group (*P* < 0.01, Figure 3(a)). However, in the other organ groups, patients who underwent operation showed no distinct differences in the overall survivals with the patients in the no surgery group (Figures 3(b)–3(e)). Our result also demonstrated that no significant difference was observed between the surgery and no surgery groups in the overall survival in final multifactor models. The overall 1-, 3-, and 5-year survival rates were 70.8% (95%CI = 48.4–84.9%), 61.2% (95%CI = 38.5–77.7%), and 55.6% (95%CI = 32.8–73.5%), respectively.

### 3.2. Survival Analysis

**Figure 4:**
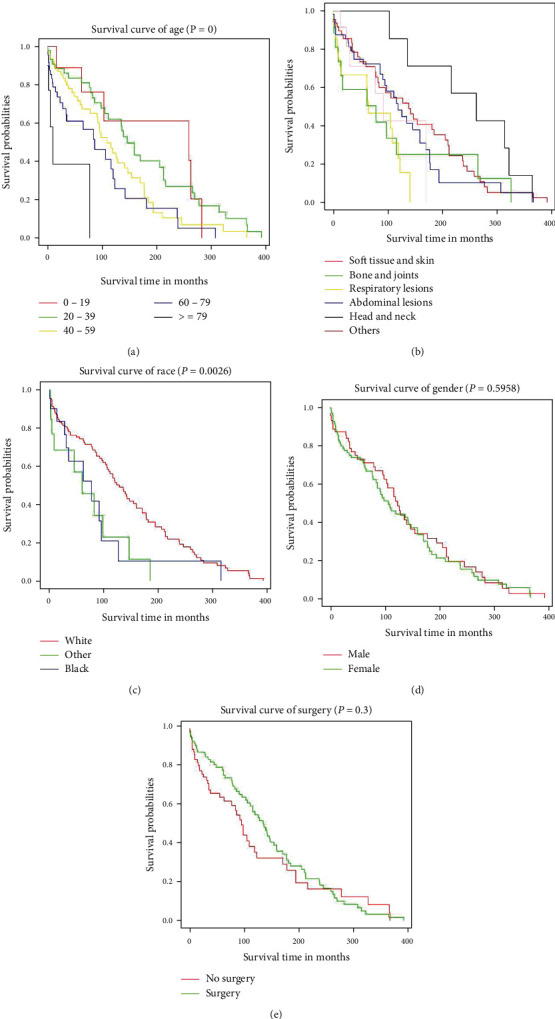
Kaplan-Meier survival curves for different variables by the use of by the log-rank test. (a) KM survival curves in age at diagnosis. (b) KM survival curves different locations. (c) KM survival curves in gender. (d) KM survival curves in surgery and no surgery groups.

**Figure 5:**
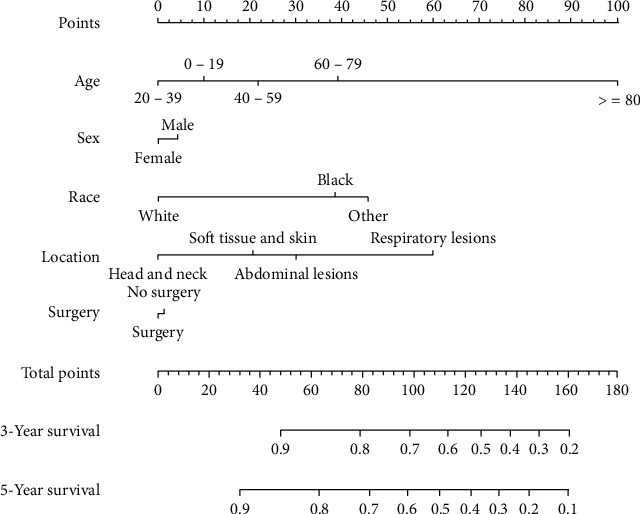
Nomograms predicting 3- and 5-year survivals of patients with epithelioid hemangioendothelioma. The C-index for survival prediction was 0.69 (95%CI = 0.635–0.744).

**Figure 6:**
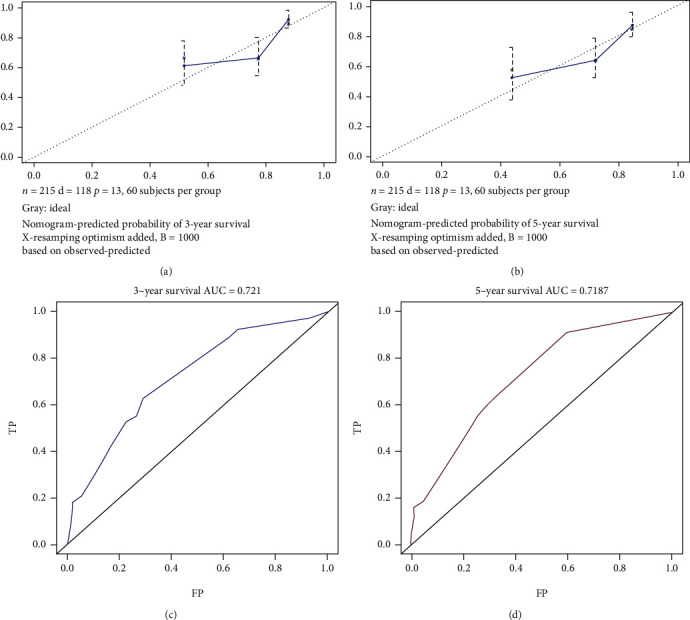
(a, b) Calibration plots of the nomogram for overall survival prediction at 3- and 5-years. (c, d) Time-dependent receiver operating characteristic curves of the nomogram for overall survival prediction at 3- and 5-years.

Owing to the lack of related data on the unknown death classification (3 patients) and race data (3 patients), only 215 patients were finally enrolled. In the univariate analysis, compared to patients aged 0–19 years, a significant difference was observed in the patients aged >80 years (HR = 8.199, 95%CI = 2.400–28.009, *P* < 0.001, Figure 4(a)). Respiratory lesions could increase the risk of death to a certain extent (HR = 2.311, 95%CI = 1.272–4.198, *P* < 0.001) compared to soft tissue and skin tumors (Table 2 and Figure 4(b)). Patients of other races (American Indian/AK Native, Asian/Pacific Islander) showed significant difference compared to those of Caucasian origin (HR = 2.322, 95%CI = 1.198–4.501, *P* = 0.013 < 0.05, Figure 4(c)). The multivariate analysis revealed that age >80 years (HR = 8.566, 95%CI = 2.320–31.626, *P* < 0.001), African American race (HR = 2.520, 95%CI = 1.309–4.853, *P* < 0.01), “American Indian/Alaska Native” or “Asian or Pacific Islander” (HR = 2.989, 95%CI = 1.498–5.964, *P* < 0.01), and respiratory tumors (HR = 2.551, 95%CI = 1.370–4.749, *P* < 0.01) were significantly associated with worse overall survival. However, no statistical difference was observed in terms of sex and treatment regimen (Table 2 and Figures 4(d) and 4(e)). According to the results obtained, a prognostic nomogram was constructed for survival at 3 and 5 years (Figure 5). The C-index for survival prediction was 0.69 (95%CI = 0.635–0.744). The calibration plots demonstrated good consistency between nomogram-predicted and actual survival (Figures 6(a) and 6(b)). Moreover, the area under the time-dependent ROC curve was 0.721 (95%CI = 0.63–0.81) and 0.719 (95%CI = 0.63–0.81) for the 3- and 5-year survival (Figures 6(c) and 6(d)). For the convenience of researchers and clinicians, we designed an online dynamic nomogram to predict the survival rate, which is available at https://plasticlz.shinyapps.io/DynNomapp/.

## 4. Discussion

EHE is a rare locally aggressive vascular neoplasm, which is considered an intermediate neoplasm between entirely benign hemangiomas and highly malignant angiosarcomas [2, 15, 16]. To date, only few case series or case reports concerning EHE have been reported. [6, 16–19] Moreover, owing to lack of high-quality clinical research, diagnosis and management of this tumor is challenging [20]. To the best of our knowledge, the data from the SEER database, which was primarily used to analyze the clinical characteristics of patients with EHE, comprise the largest published cohort of patients until now.

Some reports have suggested the IR of EHE to be less than one person per 1,000,000 person-years [21, 22]. Upon evaluating the data in the SEER database, we found similar results wherein the IR of EHE was 0.23 per 1,000,000 person-years. The IR of EHE is gradually increasing in recent years in the United States population. Some studies have found EHE to be more common in the fourth to fifth decade with rare occurrence in pediatric patients [5, 23, 24]. However, our results showed that this kind of tumor was more common in patients aged 60–79 years followed by patients aged 40–59 years. We also found that EHE was rarely seen in pediatric patients. In addition, the sex-stratified IR of this tumor was different in correlational research. Some studies suggested that EHE was more common in males [6, 19, 25]; however, Lau et al. and Stacchiotti et al. observed increased occurrence in females [5, 17, 20]. Our results demonstrated that the sex-stratified IR showed no significant difference (Table 1). Moreover, IR in our results also showed no significant difference in terms of race (Table 1). EHE occurs in various organs, such as the skin, liver, mediastinum, lung and bronchus, and oral cavity [5, 16, 20, 24]. Our study also revealed that the tumor could occur in more than 20 kinds of organs or tissues. The most common site of EHE was the soft tissue and skin (30.8%), followed by the abdomen (28%), and respiratory system (19%).

Owing to the relatively low IR of EHE, research on the overall survival rate of EHE has been limited. Based on the SEER database, our results demonstrated that the overall 1- and 5-year survival rates to be 70.8% and 55.6%, respectively. Moreover, age >80 years, African-American, and “American Indian/Alaska Native” or “Asian or Pacific Islander” race, and respiratory tumors were significantly associated with a worse overall survival. Although Lau et al. reported that male sex and a diagnosis during middle age could be related with a worse overall survival [5]. Data from the SEER database suggested that overall survival showed no statistical difference in terms of sex (*P* = 0.64). Our multivariate cox analysis did not indicate any significant difference in the overall survival between male and female patients. In our model, age >80 years was suggested to be an independent predictor for overall survival. This could be attributed to the comorbid conditions in older patients resulting in increased mortality. Moreover, tumor-related systemic symptoms, including fever, fatigue, or weight loss added to the severity of the condition in older patients [26]. Race-related overall survival rate difference may be attributed to various reasons. First, owing to the complexity of the patients' racial and ethnic backgrounds, limited relevant EHE data exist on African American and American Indian/Alaska Native patients compared to Caucasian patients [27]. Second, access to high-quality medical services for African American and American Indian/Alaska Native patients is challenging owing to economic factors. [28, 29] Lastly, differences in living habits and ethnicity between different races could contribute to the difference in the overall survival. Respiratory tumors were also associated with a worse overall survival in our model. Previous studies have reported EHE to rarely occur in the lung [5]; however, in our study, the number of EHEs in the lung and bronchus (13%) EHE was comparable among the included patients. It has been reported that once the tumor invades the bilateral lung or pleura, the life expectancy decreases significantly, even to less than 1 year [5, 17, 30]. Thus, the lesion characteristics of respiratory EHE could have contributed to the worse overall survival in our model.

Surgery is considered the primary treatment for confirmed unifocal EHE, especially in the soft tissue [20, 31]. However, in our study, fewer than half (47%) the patients underwent surgery for EHE. In the surgery group, the majority (42.6%) of the lesions were located in the soft tissue and skin. However, among the patients who underwent operations, only the patients with EHE in the soft tissue and skin group had a good overall survival. (*P* < 0.01). Moreover, in the other organ groups, patients who underwent surgery showed no significance difference in the overall survival compared to patients who did not undergo surgery. Moreover, patients in the surgery group showed no significant difference in the overall survival from those in the no surgery group in the final multifactor models (*P* = 0.3, Figure 4(e)). This could be attributed to the rare nature and highly variable clinical course of EHE. No widely accepted treatment strategy exists for EHE. Moreover, according to Kaltenmeier's report, most patients with hepatic EHE (HEHE) in the United States (93.8%) did not undergo surgery owing to the comorbidities or patient preference [21]. In our study, the most common lesion location was the liver (28.2%) and followed by the lung and bronchus (19.7%) in the patients who did not undergo surgery. The liver is reportedly the most common organ for EHE lesions [21]. While the treatment strategies for HEHE remain uncertain, some studies suggest surgery as the first treatment for HEHE [17]. However, other studies suggest liver transplantation as the best treatment option [18, 32]. The treatment for respiratory EHE also remains controversial. In unilateral focal lesions, surgery could be effective; however, in bilateral multiple nodules or pleural invasion, no effective treatment, including lung transplantation, exists [5, 30]. Owing to the low IR of EHE, the course and clinical characteristics concerning EHE remain unclear, and various treatment strategies exist. Hence, more high-quality studies defining the criteria for optimizing the selection of treatment modalities for EHE are warranted in the future.

Owing to the rare IR of EHE, certain limitations of this study should be considered. Although, the SEER database provided considerable EHE patient record, specific data, including the type of surgical resection, adequacy of the resection performed, and surgical timing, were not included. Accurate records on systemic therapy, such as chemotherapy and radiotherapy, was missing. As a retrospective database, the SEER database included certain unknown and incomplete data. Similarly, the symptoms of EHE in the SEER database were missing. Thus, the survival analysis should be interpreted with caution.

In conclusion, EHE is a relatively rare vascular tumor; however, its incidence has been increasing in recent years. It occurs principally in the soft tissue and skin, most common in patients aged 60–79 years. For patients with EHE, the age at diagnosis, race, and tumor location could affect the overall survival outcomes. The nomogram proposed in this study could estimate individualized survival for patients with EHE.

## Figures and Tables

**Table 1:** Age-adjusted incidence rates of epithelioid hemangioendothelioma.

Groups	Total number of cases	IR (per 1,000,000)	95% CI	*P* value
All years	221	0.230	0.201-0.263	—
0-19 years	10	0.036	0.017-0.066	<0.001
20-39 years	48	0.168	0.123-0.222	<0.05
40-59 years	85	0.349	0.278-0.431	<0.01
60-79 years	68	0.524	0.406-0.665	<0.001
80+ years	10	0.332	0.159-0.612	0.338
Gender	221	0.230	0.201-0.263	—
Male	98	0.212	0.172-0.259	0.545
Female	123	0.246	0.204-0.293	0.604
All races	221	0.230	0.201-0.263	—
White	179	0.233	0.200-0.270	0.949
Black	21	0.272	0.165-0.421	0.547
Others	18	0.164	0.010-0.260	0.192

**Table 2:** Univariate and multivariate cox analysis of overall survival.

Variable	Groups	Patients	Univariate analysis HR (95% CI) *P*	Multivariate analysis HR (95% CI) *P*
Age	Age 0-19	10	1 (reference) -	1 (reference) -
	Age 20-39	47	1.038 (0.431-2.500) 0.933	0.784 (0.316-1.945) 0.599
	Age 40-59	80	1.696 (0.716-4.017) 0.230	1.320 (0.538-3.240) 0.545
	Age 60-79	68	2.431 (0.999-5.914) 0.050	2.000 (0.804-4.978) 0.136
	Age 80+	10	8.199 (2.400-28.009) <0.001	8.566 (2.320-31.626) <0.01

Sex	Male	95	1 (reference) -	1 (reference) -
	Female	120	1.10404 (0.763-1.597) 0.6	0.903 (0.600-1.359) 0.625

Race	White	177	1 (reference) -	1 (reference) -
	Black	21	1.768 (0.962-3.250) 0.067	2.520 (1.309-4.853) <0.01
	Other	17	2.322 (1.198-4.501) 0.013	2.989 (1.498-5.964) <0.01

Location	Soft tissue and skin	67	1 (reference) -	1 (reference) -
	Respiratory lesions	42	2.311 (1.272-4.198) <0.001	2.551 (1.370-4.749) <0.01
	Bone and joints	18	1.418 (0.739-2.721) 0.29	1.877 (0.921-3.823) 0.083
	Abdominal lesions	61	1.205 (0.758-1.916) 0.431	1.252 (0.771-2.035) 0.364
	Head and necks	8	0.542 (0.240-1.225) 0.141	0.609 (0.254-1.459) 0.266
	Others	19	1.463 (0.605-3.533) 0.398	1.360 (0.523-3.530) 0.530

Surgery	No surgery	115	1 (reference) -	1 (reference) -
	Surgery	100	0.820 (0.563-1.193) 0.299	0.970 (0.640-1.474) 0.887

## Data Availability

The datasets generated for this study are available on request to the corresponding author.

## Abbreviations

EHE: Epithelioid hemangioendothelioma
SEER database: The surveillance, epidemiology, and end results database
ROC curve: Receiver operating characteristic curve
IR: Incidence rate
HR: Hazard ratio
C-index: concordance index.
